# Prevalence of posttraumatic stress disorder in acute trauma patients

**DOI:** 10.1097/OI9.0000000000000056

**Published:** 2020-03-03

**Authors:** Noah M. Joseph, Alex Benedick, Christopher D. Flanagan, Mary A. Breslin, Megen Simpson, Christina Ragone, Mark Kalina, Sarah B. Hendrickson, Heather A. Vallier

**Affiliations:** MetroHealth Medical Center, Cleveland, OH, affiliated with Case Western Reserve University

**Keywords:** mechanism, posttraumatic stress disorder, prevalence, PTSD, trauma

## Abstract

**Objective:**

To determine the prevalence of positive screening for posttraumatic stress disorder (PTSD) amongst trauma patients.

**Design:**

Prospective, longitudinal study.

**Setting:**

Single urban US level 1 trauma center.

**Patients and methods:**

Four hundred fifty-two adult trauma patients were administered the PTSD checklist for DSM-V (PCL-5) survey upon posthospital outpatient clinic visit. This included 300 men (66%) and 152 women with mean age 43.8 years and mean Injury Severity Score (ISS) 11.3, with 83% having fractures of the pelvis and/or extremities. Medical and injury related variables were recorded. Multivariate logistic regression analysis was performed to identify factors predictive of screening positive for PTSD.

**Main outcome measurement:**

Prevalence and risk factors for screening positive for PTSD amongst the trauma patient population.

**Results:**

Twenty-six percent of trauma patients screened positive for PTSD after mean 86 days following injury. These patients were younger (35 vs 46 years old, *P* < 0.001) and more commonly African American (56% vs 43% Caucasian, *P* < 0.001). Pedestrians struck by motor vehicles (OR 4.70, *P* = 0.040) and victims of crime (OR 4.12, *P* = 0.013) were more likely to screen positive. Psychiatric history, injury severity (ISS), and injury type did not predict positive screening.

**Conclusion:**

One-in-four patients suffering traumatic injuries screened positive for PTSD suggesting the prevalence of PTSD among trauma patients far exceeds that of the general population. Predictive factors included victims of crime and pedestrians struck by motor vehicles. Screening measures are needed in orthopaedic trauma surgery clinics to refer these at-risk patients for proper evaluation and treatment.

**Level of evidence:**

Prognostic; Level II

## 1 Introduction

Posttraumatic stress disorder (PTSD) is a well-characterized and disabling consequence of traumatic injury.^[1,2]^ While lifetime prevalence of PTSD in the general population is estimated at 6.8%, current studies suggest the prevalence is higher in patients who sustain traumatic injury with estimates reported between 13% and 51% in the orthopaedic population.^[3–7]^ While symptoms must persist for 1 month after a traumatic event and cause significant distress for a diagnosis of PTSD, the American College of Surgeons Committee on Trauma (ACS-COT) recommends that level I trauma centers screen for PTSD symptoms acutely in order to identify at-risk patients.^[3]^ With over 2 million people in the United States admitted annually for management of traumatic injuries, there is a growing need for adequate screening and early intervention for patients with symptoms of PTSD.^[8]^

It is increasingly recognized that PTSD adversely affects health outcomes, including rates of rehospitalization, return to work, activities of daily living, perception of recovery, and opioid use.^[9–12]^ Despite this negative correlation, screening for PTSD is not a routine aspect of postinjury care. Few studies to date have reported on the relationship between orthopaedic injuries and the development of PTSD.^[6,13,14]^ The purpose of this study was to determine the prevalence of positive screening for PTSD amongst trauma patients admitted to a Level I trauma center. We hypothesized that patients with lower extremity fractures and higher Injury Severity Score would be more likely to screen positive for PTSD given the degree of resulting disability.

## 2 Patients and methods

We performed an Institutional Review Board-approved longitudinal prospective study of patients who presented to the emergency department as trauma activations at an urban Level 1 Trauma Center from June 1, 2017, through September 1, 2018. This study was approved by the committee on research ethics at the institution in which the research was conducted in accordance with the Declaration of the World Medical Association (www.wma.net) and that any informed consent from human subjects was obtained as required. Of the 6688 trauma activations during this 15-month period, 945 patients (14%) were identified at first outpatient trauma or orthopaedic clinic visit for potential inclusion in this study. Four-hundred fifty-two of these patients (48%) were administered, and completed fully, the PTSD checklist for Diagnostic and Statistical Manual for mental illness Fifth edition (PCL-5) survey during their first posthospital outpatient clinic visit. The trauma center serves a greater metropolitan area of more than 2 million residents, with an even wider catchment area.

The PCL-5 is a widely validated self-report measure used in screening, diagnosing, and monitoring symptom change during and after treatment of PTSD.^[15–19]^ It consists of 20 items with a Likert scale rating from 0 (“not at all”) to 5 (“extremely”) to indicate symptom presence and severity. A score of 33 or more is considered sufficient for provisional diagnosis warranting referral to appropriate psychiatric services.^[15–19]^ All patients who screened positive for PTSD were offered appropriate ancillary services for intervention, including support groups, counseling, and outpatient psychotherapy.

Electronic medical records were used to obtain demographic and health data, including past medical history, prior history of trauma, and a history of PTSD, depression or other psychiatric illness. Specific comorbidities analyzed included cardiovascular, endocrine, neurologic, gastrointestinal, or respiratory diagnoses. History of tobacco, alcohol, and other illicit drug use was also noted. Details related to inpatient hospitalization, including injury features and treatments, were recorded. This included mechanism and type of injury, length of stay (LOS), use of mechanical ventilation, Glasgow Coma Scale (GCS) at presentation, intensive care unit (ICU) LOS, and discharge disposition. Nonorthopaedic traumatic injuries were classified by location, including head, face, chest, abdominal, neurovascular, genitourinary, burn, and soft tissue injuries. Soft tissue injuries were included if they required surgical intervention, including open fractures requiring soft tissue coverage, degloving injuries, and evacuation of hematomas. Orthopaedic injuries examined included fractures of the pelvis, acetabulum, femur, tibia, ankle, upper extremity, and open fractures. Whether a patient was a victim of a crime was also noted. Victim status was designated to patients who sustained injuries resulting from a criminal offense and included pedestrians struck by motor vehicles, assault, and non-self-inflicted gunshot wounds. Injury severity was defined by Injury Severity Score (ISS).^[20]^

### 2.1 Statistical analysis

Differences in the averages for continuous variables between subgroups were tested using Student *t* tests when the assumption of normality was satisfied. Mann–Whitney tests were used when such an assumption could not be made. Pearson Chi-square and Fisher exact tests were used to examine differences in categorical variables. Multivariate logistic regression was then performed to identify risk factors for screening positive for PTSD based on pooled demographic data, medical history, injury mechanism, and treatment-related variables. The Hosmer–Lemeshow test was used to evaluate goodness-of-fit of the regression model. Results of logistic regression analysis are expressed with use of odds ratios (OR) with their 95% confidence interval (CI). Statistical significance was set at *P* ≤ 0.05 for all outputs.

## 3 Results

Four hundred fifty-two patients were offered the PCL-5 survey; 49 (10.8%) declined or did not complete the survey in its entirety. No differences in age, sex, race, history of prior trauma, victim status, length of follow-up, or length of stay were noted between those who completed the survey and those who did not. One hundred three patients screened positive for PTSD (26%) after a mean of 86 days following injury, 97 (94%) with no prior history of PTSD. Mean PCL-5 score was 51.0 for those screening positive (versus 9.2 negative). Patients who screened positive were younger (35 vs 46 years old, *P* < 0.001), more commonly African American (56% vs 43% Caucasian, *P* < 0.001), and had fewer medical comorbidities (1.2 vs 1.6, *P* < 0.001) (Table 1).

**Table 1:**
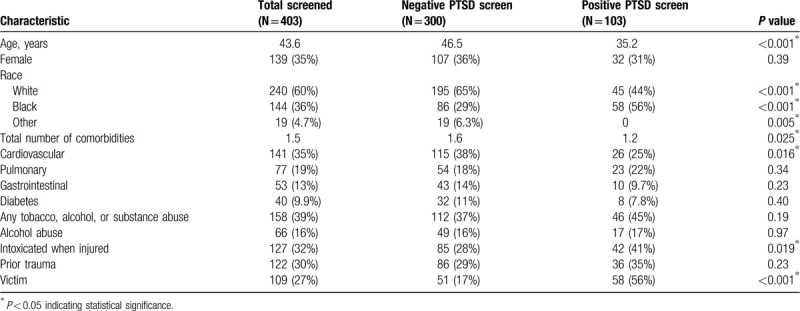
Comparison of demographic characteristics by positive versus negative screening for posttraumatic stress disorder using the PTSD checklist for DSM-V (PCL-5) survey.

There was no association between a history of substance abuse and a positive PTSD screen (44.7% vs 37.3%, *P* = 0.19). However, patients who were intoxicated at the time of injury (41% vs 28% negative screen, *P* = 0.019) and victims of crime were more likely to screen positive for PTSD (56% vs 17%, *P* < 0.001; Table 1). No significant differences between groups were seen in the frequency of pre-existing psychiatric conditions except for bipolar disorder (48% positive vs 52% negative, *P* = 0.012) (Table 2).

**Table 2:**
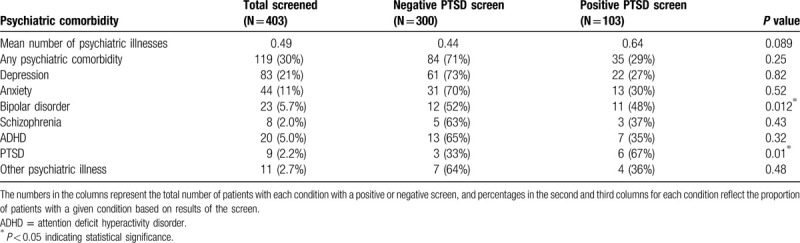
Screening results for posttraumatic stress disorder by psychiatric comorbidities.

Mechanisms of injury associated with PTSD included gunshot wounds (GSW, 53% positive vs 47%, *P* < 0.001) and pedestrians struck by motor vehicles (59% vs 41%, *P* = 0.003; Table 3). No differences between groups were seen in the frequency of different mechanisms of injury, including motor vehicle collision, motorcycle or ATV collision, stabbing or physical assault, sports or bicycle injury, crush or work injury, burn or blast injury, or other mechanisms of injury. There was no difference in PCL-5 score based on ISS (10.5 for positive vs 11.6, *P* = 0.24; Table 4).

**Table 3:**
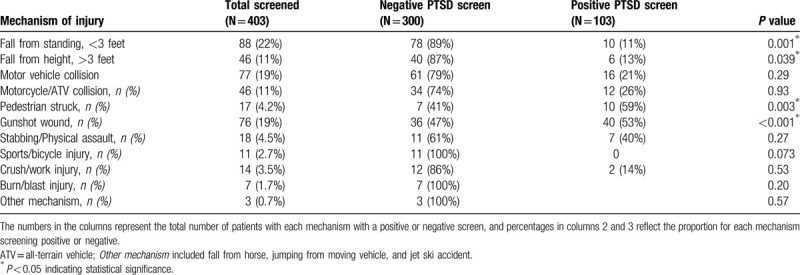
Screening results for PTSD by mechanism of injury.

**Table 4:**
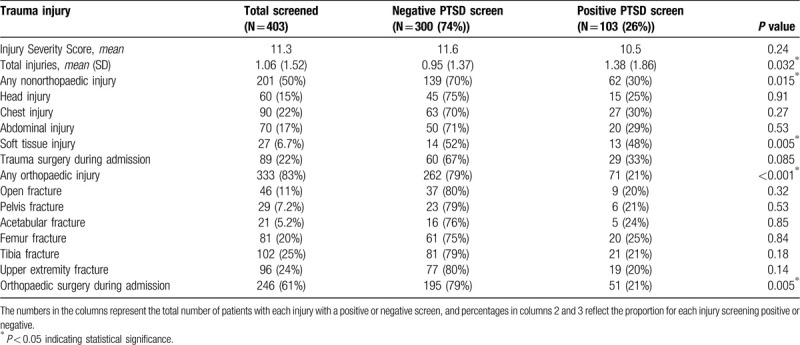
Screening results for PTSD based on injury types.

Sixty-two patients in the positive PTSD screening cohort sustained nonorthopaedic traumatic injuries, with 28% requiring surgery during admission. Those who screened positive for PTSD sustained more total traumatic injuries (1.38 vs 0.95, *P* = 0.032; Table 4). They were also more likely to have sustained soft tissue injuries (13% positive vs 5% negative, *P* = 0.005). No other association was found between injury type and positive PTSD screen. This included orthopaedic injury and all fracture types analyzed (Table 4). Variables related to hospital stay were not related to PCL-5 score, including length of hospital stay, ICU stay, days intubated, or discharge disposition.

Multivariate logistic regression analysis showed that pedestrians struck by motor vehicles (OR 4.70, *P* = 0.040) and victims of crime (OR 4.12, *P* = 0.013) were more likely to screen positive for PTSD. Neither age, psychiatric history, nor injury type or severity were associated with screening positive for PTSD (Table 5).

**Table 5:**
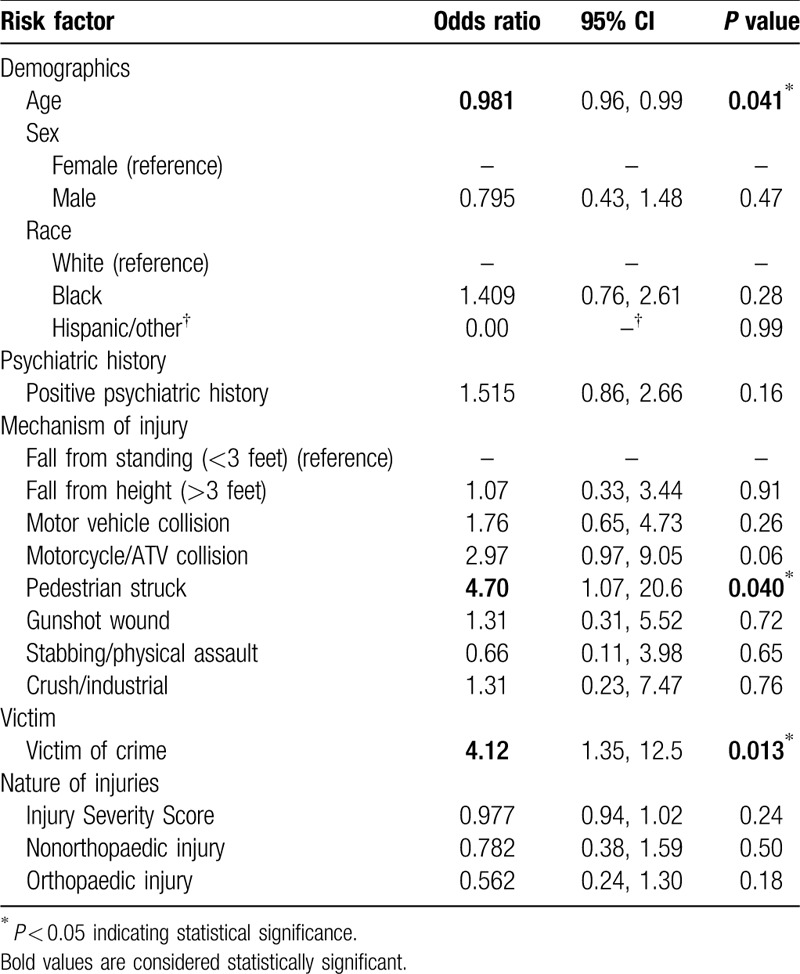
Multivariate logistic regression analysis of potential risk factors for screening positive for PTSD.

## 4 Discussion

PTSD is a debilitating mental illness that negatively impacts health outcomes and quality of life amongst the trauma population.^[21,22]^ Nevertheless, screening for PTSD is not routinely performed in orthopaedic trauma clinics. We administered the PCL-5 questionnaire at first posthospital outpatient visit. We identified that 26% screened positive for PTSD, a larger proportion of which were younger, African American and sustained GSWs. Multivariate regression analysis showed that victims of crime, including pedestrians struck by motor vehicles, were 4 times more likely to screen positive for PTSD. These findings offer insight into those most vulnerable to developing PTSD after trauma.

Previous work reporting the prevalence of PTSD following trauma has shown considerable variation secondary to inconsistencies in methodology and study populations.^[3–10]^ In a recent meta-analysis, Muscatelli et al^[6]^ identified just 11 prior studies from 1991 to 2014 reporting prevalence of PTSD in trauma patients. Amongst these, the reported prevalence of PTSD varied considerably between 13.3 and 50.9% for both military and civilian populations with sample sizes ranging from 48 to 580 subjects. Methodology also varied with 8 different metrics utilized to screen for PTSD. Of those studies, Starr et al^[7]^ described the largest sample of orthopaedic trauma patients screened for PTSD (N = 580) and identified more than half met diagnostic criteria for PTSD 1 year following injury. However, they did not include any GSW patients in their series, nor were they able to identify demographic or injury-related risk factors for those who screened positive for PTSD. In comparison, our cohort of 452 trauma patients would represent the second largest to be described using a well-validated screening and diagnostic tool in the PCL-5. Furthermore, our results illustrate a relationship between mechanism of injury and the development of PTSD offering the potential to effectively target and refer vulnerable patient populations.

In our cohort we found patients who screened positive for PTSD were more commonly African American and 10 years younger than those who screened negative. Previous studies have consistently reported younger age to be a predictor of PTSD due to the propensity for younger individuals to be involved in assaultive trauma.^[12,23–25]^ While ethnicity has been reported in some studies to be associated with the development of PTSD, it has been reported to be insignificant in others.^[12]^ This may be attributed to differences in patient population as well as types of analyses.

Injury type, mechanism, and severity have all been linked to the development of posttraumatic psychiatric conditions.^[26–30]^ The effect of any one of these factors in isolation is difficult to ascertain as their effect is likely combinatorial. We found that victims of crime, including pedestrians struck by motor vehicles, were more likely to screen positive for PTSD. Neither injury severity nor injury type was predictive of developing PTSD. These results illustrate that mechanism of injury may play a more important role in the development of PTSD. An understanding of these risk factors in the development of PTSD can alert providers to screen and refer vulnerable patients for appropriate psychiatric treatment.

Prior work has implicated orthopaedic injury and injury severity in the development of PTSD.^[3–7]^ It has been postulated that injury type and severity may be representative of the degree of pain and functional impairment following an injury or treatment process.^[31–36]^ Uncontrolled pain has been shown to disturb early rehabilitation and can lead to the persistence of functional impairment, which can in turn lead to chronic disability and psychological illness.^[31,32]^ Patients with lower extremity fractures or multiple extremity fractures tend to have longer periods of functional impairment secondary to limited weight bearing compared to single fractures or upper extremity fractures.^[10]^ This prolonged period of disability has been reported to result in higher rates of psychiatric illness in other studies.^[4,6,10–12]^ Despite these previous reports, we saw no association between ISS, fracture type, or location and PTSD. Thus, while these factors may play a key role in the development of other psychiatric illnesses such as depression, they appear less related to the development of PTSD.

Many psychosocial issues may predispose trauma patients to develop PTSD. Pre-existing psychiatric illness and socioeconomic status are strongly associated with increased disability in patients recovering from musculoskeletal trauma.^[24,37–39]^ This has been linked to heightened perception of pain, coping difficulties, and availability of psychosocial support.^[38]^ We found no association between prior psychiatric illness and positive PTSD screening among our sample. Further study is warranted to further elucidate this relationship.

The results of our study should be considered in light of some limitations. Most notably, screening was performed at outpatient clinics for general and orthopaedic trauma at first posthospital visit. As a result, we were limited by those patients who did not return to clinic, were lost to follow-up, or who declined to complete the survey. In addition, our data related to comorbid conditions, including history of mental illness and substance abuse, was abstracted from clinical records. Therefore, we may be underestimating the frequency of these conditions in this population secondary to patient failure to report and physician failure to assess or document associated psychiatric history.

Lastly, we defined positive screening for PTSD as a PCL-5 score of 33 or more. Psychometrics for the PCL-5 validated against the Clinician administered PTSD scale for DSM-V (CAPS-5), which is considered the gold-standard for diagnosing PTSD, suggest that cutoff is diagnostic.^[15–19]^ However, those who scored slightly below this threshold may still suffer significant psychological distress or go on to develop clinically diagnosed PTSD. Therefore, we may be underestimating the prevalence of PTSD in this population.

In conclusion, we found that a quarter of trauma patients screened positive for PTSD. While prior work has described a strong association between injury type (i.e., lower extremity fracture), severity (i.e., ISS), and pre-existing mental illness (i.e., depression) in the development of PTSD, our results indicate that mechanism of injury may play a more important role.^[4,6,7]^ To our knowledge, this finding is novel. Because acute intervention, in the form of pharmacotherapy and psychotherapy, is highly effective in treating and preventing chronic disability, future studies aimed at enhanced risk factor identification for PTSD and evaluating the efficacy of psychiatric referral programs have the potential to guide future management of survivors of trauma.^[40–44]^ By better identifying patients at risk of developing PTSD, education and resources can be provided at the time of hospitalization or outpatient follow-up to ultimately reduce rates of PTSD in this vulnerable population.
